# A two-dimensional copper(I) coordination polymer based on 1-[2-(cyclo­hexyl­sulfan­yl)eth­yl]pyridin-2(1*H*)-one

**DOI:** 10.1107/S2056989017015377

**Published:** 2017-10-27

**Authors:** Hyunjin Park, Jineun Kim, Hansu Im, Tae Ho Kim

**Affiliations:** a Department of Chemistry (BK21 plus) and Research Institute of Natural Sciences, Gyeongsang National University, Jinju 52828, Republic of Korea

**Keywords:** crystal structure, coordination polymer, copper(I) iodide, S/O donors

## Abstract

The reaction of copper(I) iodide with 1-[2-(cyclo­hexyl­sulfan­yl)eth­yl]pyridin-2(1*H*)-one (*L*, C_13_H_19_NOS) in aceto­nitrile/di­chloro­methane results in a crystalline coordination polymer, namely poly[bis­{μ_2_-1-[2-(cyclo­hexyl­sulfan­yl)eth­yl]pyridin-2(1*H*)-one}tetra-μ_3_-iodido­tetra­copper(I)], [Cu_4_I_4_ *L*_2_]_*n*_. The asymmetric unit comprises two ligand mol­ecules, four copper(I) ions and four iodide ions. Inter­estingly, the O atoms are bound to the soft copper(I) ions. The stair-step clusters of Cu and I atoms in the asymmetric unit are linked repeatedly, giving rise to infinite chains along [100]. Neighbouring infinite chains are linked through the *L* mol­ecules, forming a two-dimensional brick-wall structure. These two-dimensional networks are stacked alternately along [001]. Additionally, there are inter­molecular C—H⋯I hydrogen bonds and C—H⋯π inter­actions between the ligands.

## Abstract 2

The structure of the title copper(I) coordination polymer is reported. One Cu^I^ atom is surrounded by three μ_3_-iodide anions and one S atom, while the other is coordinated by three μ_3_-iodide ions an O atom. In the crystal, there are inter­molecular C—H⋯I hydrogen bonds and C—H⋯π inter­actions between ligands. The packing generates a two-dimensional brick-wall structure.

## Chemical context   

Copper(I) complexes have been studied continuously over several decades because of their potential applications as sensors, catalysts, and gas storage materials (Lin *et al.*, 2016 ▸; Ananthnag *et al.*, 2015 ▸; Pal *et al.*, 2015 ▸). They exhibit a variety of structures, photoluminescence, and other physical properties as a result of the *d* ^10^ electron configuration of Cu^I^ (Peng *et al.*, 2010 ▸; Ford *et al.*, 1999 ▸; Kobayashi & Kato, 2017 ▸). In addition, the arrangement of donor atoms in the ligands may affect both the structures of the complexes and their physical properties. Copper(I) complexes of flexible ligands with N/S donor atoms have been studied (Jeon *et al.*, 2014 ▸; Cho *et al.*, 2015 ▸). Mechanochromism, vapochromism and solvatochromism of such complexes have also been reported (Kwon *et al.*, 2017 ▸; Kang *et al.*, 2015 ▸; Kim *et al.*, 2013 ▸). Herein we describe the synthesis and crystal structure of a copper(I) complex [Cu_4_I_4_ *L*_2_]_*n*_ of *L* (C_13_H_19_NOS) with O/S donor atoms. Cu^I^—O bonds have been reported previously in copper(I) coordination polymers with phosphine ligands (Darensbourg *et al.*, 1998 ▸) but those with an O/S donor ligand set are unique as far as we know.

## Structural commentary   

**Figure 1:**
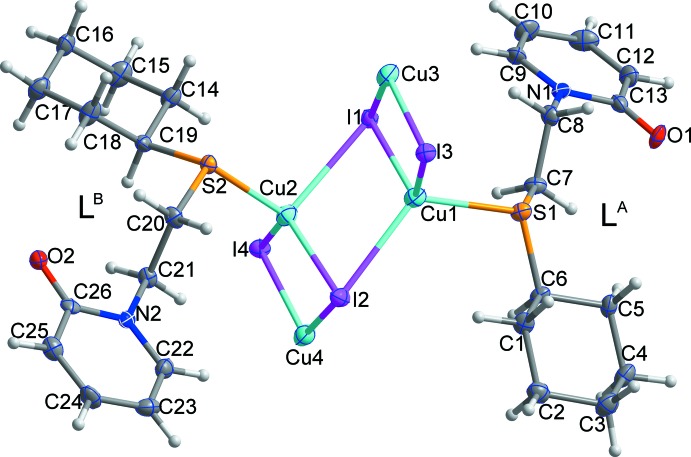
The mol­ecular structure of the title compound, with the atom labelling and displacement ellipsoids drawn at the 50% probability level. H atoms are shown as small spheres of arbitrary radius.

The asymmetric unit of the title compound, [Cu_4_I_4_ *L*_2_]_*n*_, comprises four copper(I) ions, four μ_3_-iodide ions, and two *L* ligands as shown in Fig. 1 ▸. In L^A^ (identified by S1) and L^B^ (identified by S2), the pyridyl and cyclo­hexyl rings are in *anti* and *gauche* conformations with torsion angles of −154.7 (6)° [C6—S1—C7—C8] and 62.3 (7)° [C19—S2—C20—C21], respectively. All of the Cu^I^ atoms (Cu1–Cu4) have distorted tetra­hedral coordination geometries. The Cu1 and Cu2 atoms are bound by three μ_3_-iodide anions and one S atom, while Cu3 and Cu4 are coordinated by three μ_3_-iodide ions and one O atom. The ranges of inter­atomic distances in the title compound are 2.7082 (15)–2.7444 (14) Å, 2.297 (2)–2.314 (2) Å, 2.6210 (12)–2.7230 (12) Å, and 2.071 (6)–2.087 (6) Å for Cu—Cu, Cu—S, Cu—I, and Cu—O, respectively (Table 1 ▸). Inter­estingly, the O atoms bind to the soft copper(I) cations, implying that the carbonyl O atoms conjugated with pyridyl rings are softer than the hard, ether-like O atoms.
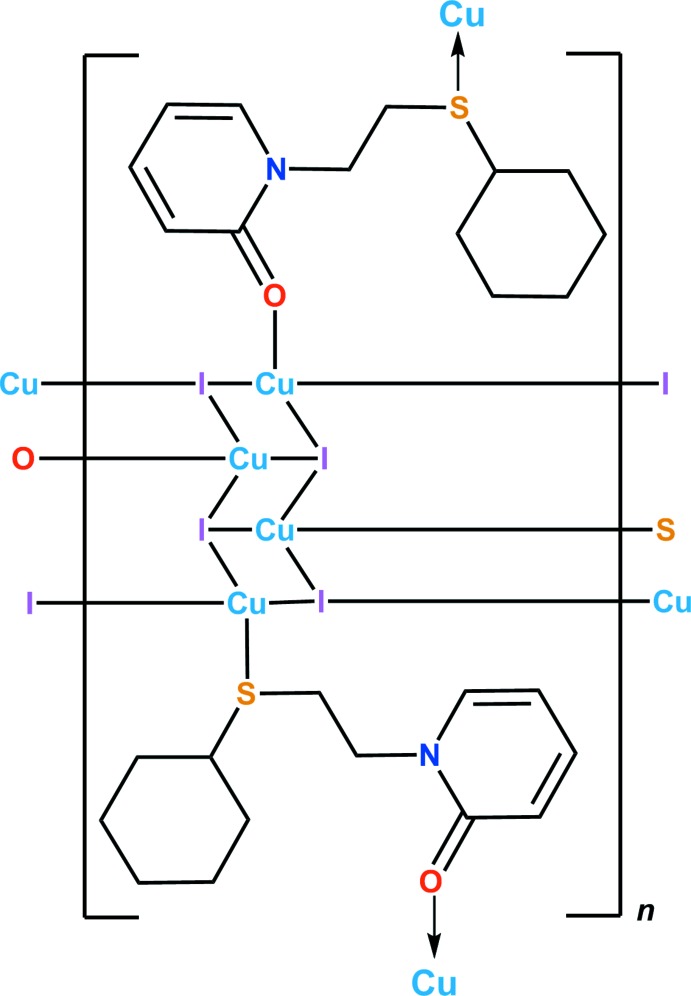


## Supra­molecular features   

**Figure 2:**
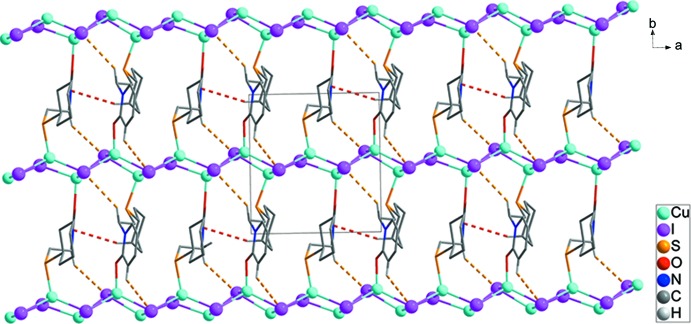
A packing diagram showing the inter­molecular C—H⋯I hydrogen bonds (yellow dashed lines) and C—H⋯π inter­actions (red dashed lines) between ligands. H atoms have been omitted for clarity.

**Figure 3:**
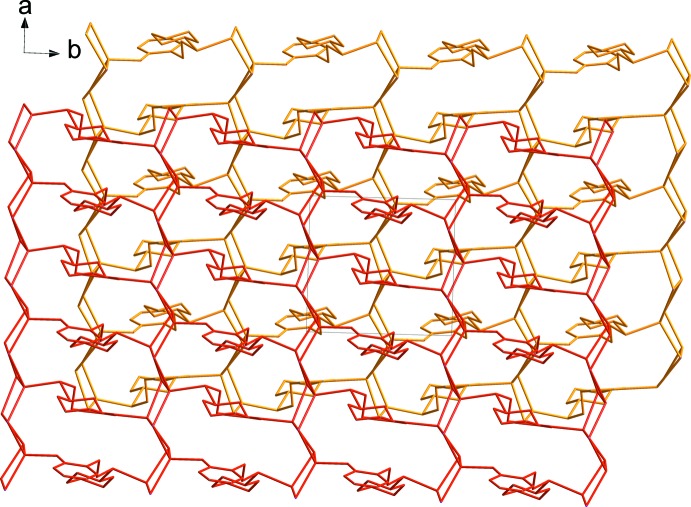
The two-dimensional brick-wall networks are stacked in an ⋯*ababab*⋯ fashion along [001]. All H atoms have been omitted for clarity.

The step-like clusters of Cu and I atoms in the asymmetric unit are linked repeatedly, generating infinite chains along [100]. Neighbouring infinite chains are linked by the *L* mol­ecules, forming a two-dimensional brick-wall structure parallel to (001) as shown in Fig. 2 ▸ (Tzeng & Chang, 2009 ▸). Yellow dashed lines display inter­molecular C8—H8*A*⋯I4^ii^, C12—H12⋯I1^iii^ and C21—H21*B*⋯I3^iv^ [H⋯I = 3.26, 3.30, and 3.08 Å, respectively] hydrogen bonds between ligands. Red dashed lines display inter­molecular C5—H5*A*⋯*Cg*1^v^ [H⋯*Cg*1=3.00 Å] inter­actions between the ligands (Fig. 2 ▸ and Table 2 ▸). The two-dimensional brick-wall networks are stacked in an ⋯*ababab*⋯ fashion along [001] (Fig. 3 ▸).

## Database survey   

Syntheses and properties of the copper(I) complexes of N/S mixed donor atom ligands have been reported (Jeon *et al.*, 2014 ▸; Cho *et al.*, 2015 ▸). Copper(I) complexes of N/S mixed-donor atom ligands with cyclo­hexyl group have also been reported (Park *et al.*, 2016 ▸, 2017 ▸). In addition, a database search (CSD Version 5.27, last update February 2017; Groom *et al.*, 2016 ▸) showed the crystal structures of three complexes with infinite stair-step (CuI)_*n*_ cluster units (Jess *et al.*, 2007 ▸; Jess & Näther, 2004 ▸; Graham *et al.*, 2000 ▸).

## Synthesis and crystallization   

**Synthesis of 1-[2-(cyclo­hexyl­sulfan­yl)eth­yl]pyridin-2(1** ***H*** **)-one (** ***L*** **)**

Thionyl chloride (2.38 g, 20.0 mmol) was added dropwise to 2-(cyclo­hexyl­thio)­ethanol (3.21 g, 20 mmol) in chloro­form. The mixture was stirred under reflux for 1 h then cooled to 253 K. Chloro­form was removed, yielding crude 2-chloro­ethyl­cyclo­hexyl­sulfide. 2-Hy­droxy­pyridine (1.90 g, 20 mmol) and potassium hydroxide (1.12 g, 20 mmol) were dissolved in 10 ml of tetra­hydro­furan and 5 ml of water, and then the solution was added dropwise to the crude chloride. The solution was refluxed for 24 h and cooled. The crude product was extracted by di­chloro­methane. The di­chloro­methane layer was dried with anhydrous Na_2_SO_4_, and evaporated to give a crude oil. Column chromatography (silica gel, MeCOOEt/*n*–C_6_H_14_ = 30/70 (*v*/*v*), *R*_f_ = 0.28) (Park *et al.*, 2016 ▸). ^1^H NMR (300 MHz, CDCl_3_): 7.28 (*dd*, 2H, py), 6.52 (*d*, H, py), 6.11 (*d*, H, py), 4.01 (*t*, 2H, NCH_2_), 2.85 (*t*, 2H, CH_2_S), 2.51 (*d*, H, SCH), 2.00–1.13 [*m*, 10H, (CH_2_)_5_]; ^13^C NMR (39.51 MHz, DMSO): 161.33, 140.03, 139.52, 119.40, 104.86, 49.21, 42.48, 33.15 27.71, 25.44, 25.29.

**Preparation of [Cu_4_I_4_** ***L*** **_2_]** ***_n_***

A di­chloro­methane (5 ml) solution of *L* (0.006 g, 0.025 mmol) was allowed to mix with an aceto­nitrile (5 ml) solution of CuI (0.010 g, 0.053 mmol). The colourless precipitate was filtered and washed with a diethyl ether/aceto­nitrile (5/1 *v*/*v*) solution. Single crystals suitable for X-ray analysis were obtained by slow evaporation of di­chloro­methane from the reaction mixture.

## Refinement   

Crystal data, data collection and structure refinement details are summarized in Table 3 ▸. All H atoms were positioned geometrically and refined using a riding model, with C—H = 0.95 Å and *U*_iso_(H) = 1.2*U*_eq_(C) for aromatic C—H groups, C—H = 0.99 Å and *U*_iso_(H) = 1.2*U*_eq_(C) for CH_2_ groups, and C—H = 1.00 Å and *U*_iso_(H) = 1.2*U*_eq_(C) for Csp^3^—H groups.

## Supplementary Material

Crystal structure: contains datablock(s) I, New_Global_Publ_Block. DOI: 10.1107/S2056989017015377/sj5538sup1.cif

Structure factors: contains datablock(s) I. DOI: 10.1107/S2056989017015377/sj5538Isup2.hkl

CCDC reference: 1581394

Additional supporting information:  crystallographic information; 3D view; checkCIF report

## Figures and Tables

**Table 1:** Selected bond lengths (Å)

Cu1—S1	2.314 (2)	Cu2—I1	2.7230 (12)
Cu1—I1	2.6467 (12)	Cu3—O1^i^	2.087 (6)
Cu1—I2	2.6669 (12)	Cu3—I3	2.6210 (12)
Cu1—I3	2.6939 (12)	Cu3—I1	2.6458 (12)
Cu1—Cu3	2.7444 (14)	Cu3—I4^ii^	2.6833 (12)
Cu2—S2	2.297 (2)	Cu4—O2^iii^	2.071 (6)
Cu2—I4	2.6256 (12)	Cu4—I4	2.6412 (13)
Cu2—I2	2.6544 (12)	Cu4—I3^iv^	2.6800 (13)
Cu2—Cu4	2.7082 (15)	Cu4—I2	2.7084 (13)

Symmetry codes: (i) 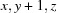; (ii) 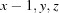; (iii) 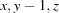; (iv) 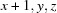.

**Table 2:** Hydrogen-bond geometry (Å, °) *Cg*1 is the centroid of the N2/C22–C26 ring

*D*—H⋯*A*	*D*—H	H⋯*A*	*D*⋯*A*	*D*—H⋯*A*
C8—H8*A*⋯I4^ii^	0.99	3.26	4.107 (8)	145
C12—H12⋯I1^iii^	0.95	3.30	3.899 (8)	123
C21—H21*B*⋯I3^iv^	0.99	3.08	3.923 (8)	144
C5—H5*A*⋯*Cg*1^v^	0.99	3.00	3.948 (9)	162

Symmetry codes: (ii) 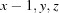; (iii) 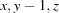; (iv) 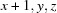; (v) 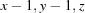.

**Table 3:** Experimental details

Crystal data	
Chemical formula	[Cu_4_I_4_(C_13_H_19_NOS)_2_]
*M*_r_	1236.46
Crystal system, space group	Monoclinic, *P*2_1_
Temperature (K)	173
*a*, *b*, *c* (Å)	8.5922 (3), 9.1285 (3), 21.5629 (6)
β (°)	96.754 (1)
*V* (Å^3^)	1679.53 (9)
*Z*	2
Radiation type	Mo *K*α
μ (mm^−1^)	6.33
Crystal size (mm)	0.35 × 0.27 × 0.03

Data collection	
Diffractometer	Bruker APEXII CCD
Absorption correction	Multi-scan (*SADABS*; Bruker, 2014 ▸)
*T*_min_, *T*_max_	0.402, 0.746
No. of measured, independent and observed [*I* > 2σ(*I*)] reflections	29149, 7588, 7376
*R*_int_	0.047
(sin θ/λ)_max_ (Å^−1^)	0.649

Refinement	
*R*[*F* ^2^ > 2σ(*F* ^2^)], *wR*(*F* ^2^), *S*	0.031, 0.072, 1.10
No. of reflections	7588
No. of parameters	362
No. of restraints	1
H-atom treatment	H-atom parameters constrained
Δρ_max_, Δρ_min_ (e Å^−3^)	2.35, −0.84
Absolute structure	Refined as an inversion twin.
Absolute structure parameter	0.07 (3)

Computer programs: *APEX2* and *SAINT* (Bruker, 2014 ▸), *SHELXS97* and *SHELXTL* (Sheldrick, 2008 ▸), *SHELXL2014* (Sheldrick, 2015 ▸), *DIAMOND* (Brandenburg, 2010 ▸) and *publCIF* (Westrip, 2010 ▸).

## supplementary crystallographic information

### Crystal data

**Table:**

[Cu_4_I_4_(C_13_H_19_NOS)_2_]	*F*(000) = 1168
*M**_r_* = 1236.46	*D*_x_ = 2.445 Mg m^−^^3^
Monoclinic, *P*2_1_	Mo *K*α radiation, λ = 0.71073 Å
*a* = 8.5922 (3) Å	Cell parameters from 9858 reflections
*b* = 9.1285 (3) Å	θ = 2.4–27.5°
*c* = 21.5629 (6) Å	µ = 6.33 mm^−^^1^
β = 96.754 (1)°	*T* = 173 K
*V* = 1679.53 (9) Å^3^	Plate, colourless
*Z* = 2	0.35 × 0.27 × 0.03 mm

### Data collection

**Table:**

Bruker APEXII CCD diffractometer	7376 reflections with *I* > 2σ(*I*)
φ and ω scans	*R*_int_ = 0.047
Absorption correction: multi-scan (SADABS; Bruker, 2014)	θ_max_ = 27.5°, θ_min_ = 1.0°
*T*_min_ = 0.402, *T*_max_ = 0.746	*h* = −11→10
29149 measured reflections	*k* = −11→11
7588 independent reflections	*l* = −27→27

### Refinement

**Table:**

Refinement on *F*^2^	Hydrogen site location: inferred from neighbouring sites
Least-squares matrix: full	H-atom parameters constrained
*R*[*F*^2^ > 2σ(*F*^2^)] = 0.031	*w* = 1/[σ^2^(*F*_o_^2^) + (0.0224*P*)^2^ + 3.3344*P*] where *P* = (*F*_o_^2^ + 2*F*_c_^2^)/3
*wR*(*F*^2^) = 0.072	(Δ/σ)_max_ = 0.001
*S* = 1.10	Δρ_max_ = 2.35 e Å^−^^3^
7588 reflections	Δρ_min_ = −0.84 e Å^−^^3^
362 parameters	Absolute structure: Refined as an inversion twin.
1 restraint	Absolute structure parameter: 0.07 (3)

### Special details

**Table:**

Geometry. All esds (except the esd in the dihedral angle between two l.s. planes) are estimated using the full covariance matrix. The cell esds are taken into account individually in the estimation of esds in distances, angles and torsion angles; correlations between esds in cell parameters are only used when they are defined by crystal symmetry. An approximate (isotropic) treatment of cell esds is used for estimating esds involving l.s. planes.
Refinement. Refined as a 2-component inversion twin

### Fractional atomic coordinates and isotropic or equivalent isotropic displacement parameters (Å^2^)

**Table:**

	*x*	*y*	*z*	*U*_iso_*/*U*_eq_	
Cu1	0.14206 (13)	0.42954 (11)	0.71506 (5)	0.0218 (2)	
Cu2	0.46250 (13)	0.57157 (12)	0.77959 (5)	0.0208 (2)	
Cu3	−0.03849 (13)	0.58815 (13)	0.78775 (5)	0.0243 (2)	
Cu4	0.66577 (14)	0.42199 (11)	0.71635 (5)	0.0259 (3)	
I1	0.22291 (6)	0.45860 (6)	0.83671 (2)	0.01699 (11)	
I2	0.38662 (6)	0.51673 (8)	0.65893 (3)	0.01980 (13)	
I3	−0.11520 (6)	0.57738 (7)	0.66655 (2)	0.01633 (12)	
I4	0.72270 (6)	0.46385 (6)	0.83810 (2)	0.01796 (12)	
S1	0.0444 (2)	0.1958 (2)	0.69520 (9)	0.0164 (4)	
S2	0.4169 (2)	0.8155 (2)	0.79653 (9)	0.0148 (4)	
O1	−0.0391 (7)	−0.1909 (6)	0.8126 (3)	0.0204 (13)	
O2	0.6473 (7)	1.1978 (6)	0.7028 (3)	0.0186 (13)	
N1	0.0334 (8)	0.0164 (9)	0.8668 (3)	0.0157 (14)	
N2	0.6273 (7)	0.9801 (8)	0.6501 (3)	0.0148 (14)	
C1	0.1061 (10)	0.2002 (9)	0.5753 (4)	0.0179 (17)	
H1A	−0.0086	0.2068	0.5636	0.022*	
H1B	0.1467	0.3007	0.5835	0.022*	
C2	0.1812 (11)	0.1344 (10)	0.5219 (4)	0.0240 (19)	
H2A	0.2965	0.1348	0.5323	0.029*	
H2B	0.1551	0.1948	0.4840	0.029*	
C3	0.1253 (11)	−0.0219 (10)	0.5087 (4)	0.0264 (19)	
H3A	0.1808	−0.0644	0.4752	0.032*	
H3B	0.0116	−0.0217	0.4941	0.032*	
C4	0.1567 (11)	−0.1158 (10)	0.5679 (4)	0.0230 (19)	
H4A	0.1128	−0.2151	0.5594	0.028*	
H4B	0.2712	−0.1257	0.5794	0.028*	
C5	0.0835 (10)	−0.0479 (9)	0.6222 (4)	0.0197 (17)	
H5A	−0.0321	−0.0486	0.6126	0.024*	
H5B	0.1115	−0.1074	0.6602	0.024*	
C6	0.1398 (9)	0.1084 (8)	0.6341 (3)	0.0128 (15)	
H6	0.2555	0.1072	0.6470	0.015*	
C7	0.1085 (9)	0.0872 (10)	0.7639 (4)	0.0168 (15)	
H7A	0.1198	−0.0167	0.7520	0.020*	
H7B	0.2115	0.1227	0.7836	0.020*	
C8	−0.0127 (10)	0.1007 (9)	0.8096 (4)	0.0178 (16)	
H8A	−0.0249	0.2051	0.8205	0.021*	
H8B	−0.1151	0.0648	0.7896	0.021*	
C9	0.0908 (10)	0.0879 (10)	0.9203 (4)	0.0204 (17)	
H9	0.1047	0.1911	0.9199	0.024*	
C10	0.1273 (11)	0.0125 (11)	0.9735 (4)	0.026 (2)	
H10	0.1665	0.0632	1.0106	0.031*	
C11	0.1087 (11)	−0.1392 (10)	0.9752 (4)	0.026 (2)	
H11	0.1344	−0.1920	1.0130	0.031*	
C12	0.0525 (10)	−0.2107 (9)	0.9212 (4)	0.0213 (19)	
H12	0.0394	−0.3140	0.9221	0.026*	
C13	0.0133 (10)	−0.1352 (9)	0.8641 (4)	0.0171 (17)	
C14	0.5272 (11)	0.8042 (9)	0.9183 (4)	0.0205 (18)	
H14A	0.5473	0.6987	0.9122	0.025*	
H14B	0.4164	0.8157	0.9258	0.025*	
C15	0.6351 (11)	0.8607 (10)	0.9755 (4)	0.025 (2)	
H15A	0.6108	0.8087	1.0135	0.030*	
H15B	0.7458	0.8407	0.9699	0.030*	
C16	0.6116 (12)	1.0253 (10)	0.9833 (4)	0.025 (2)	
H16A	0.5038	1.0435	0.9934	0.030*	
H16B	0.6855	1.0611	1.0188	0.030*	
C17	0.6371 (11)	1.1094 (10)	0.9256 (4)	0.0240 (19)	
H17A	0.7483	1.1007	0.9183	0.029*	
H17B	0.6146	1.2143	0.9320	0.029*	
C18	0.5324 (12)	1.0535 (9)	0.8678 (4)	0.023 (2)	
H18A	0.4211	1.0728	0.8728	0.028*	
H18B	0.5579	1.1067	0.8303	0.028*	
C19	0.5564 (10)	0.8896 (9)	0.8591 (4)	0.0174 (17)	
H19	0.6658	0.8711	0.8495	0.021*	
C20	0.4537 (9)	0.9200 (9)	0.7285 (4)	0.0182 (17)	
H20A	0.3767	0.8904	0.6930	0.022*	
H20B	0.4361	1.0250	0.7368	0.022*	
C21	0.6182 (9)	0.9018 (9)	0.7097 (4)	0.0150 (16)	
H21A	0.6964	0.9424	0.7426	0.018*	
H21B	0.6414	0.7966	0.7046	0.018*	
C22	0.6172 (10)	0.9017 (10)	0.5954 (4)	0.0208 (18)	
H22	0.6056	0.7983	0.5967	0.025*	
C23	0.6234 (10)	0.9677 (11)	0.5402 (4)	0.0247 (19)	
H23	0.6164	0.9119	0.5028	0.030*	
C24	0.6405 (10)	1.1220 (10)	0.5384 (4)	0.024 (2)	
H24	0.6450	1.1701	0.4996	0.029*	
C25	0.6506 (10)	1.2014 (10)	0.5922 (4)	0.0204 (18)	
H25	0.6641	1.3046	0.5905	0.024*	
C26	0.6411 (9)	1.1320 (9)	0.6513 (4)	0.0161 (17)	

### Atomic displacement parameters (Å^2^)

**Table:**

	*U*^11^	*U*^22^	*U*^33^	*U*^12^	*U*^13^	*U*^23^
Cu1	0.0283 (6)	0.0185 (5)	0.0189 (5)	0.0022 (4)	0.0032 (4)	−0.0009 (4)
Cu2	0.0242 (6)	0.0175 (5)	0.0204 (5)	0.0019 (5)	0.0022 (4)	0.0004 (4)
Cu3	0.0324 (6)	0.0188 (5)	0.0213 (6)	0.0011 (5)	0.0015 (5)	−0.0030 (5)
Cu4	0.0350 (7)	0.0201 (5)	0.0231 (6)	−0.0005 (5)	0.0051 (5)	−0.0035 (4)
I1	0.0184 (2)	0.0185 (2)	0.0138 (2)	−0.0043 (2)	0.00072 (19)	0.0002 (2)
I2	0.0172 (3)	0.0261 (2)	0.0159 (3)	−0.0023 (2)	0.0014 (2)	−0.0020 (2)
I3	0.0174 (3)	0.0159 (2)	0.0156 (2)	0.0027 (2)	0.0016 (2)	0.0016 (2)
I4	0.0189 (2)	0.0196 (2)	0.0154 (2)	0.0012 (2)	0.00160 (19)	0.0013 (2)
S1	0.0189 (10)	0.0158 (9)	0.0146 (10)	0.0038 (8)	0.0018 (8)	0.0016 (7)
S2	0.0158 (10)	0.0137 (8)	0.0148 (10)	0.0001 (7)	0.0010 (8)	0.0008 (7)
O1	0.032 (4)	0.014 (3)	0.014 (3)	−0.003 (2)	−0.003 (3)	−0.004 (2)
O2	0.019 (3)	0.016 (3)	0.021 (3)	−0.002 (2)	0.005 (2)	−0.001 (3)
N1	0.019 (3)	0.015 (3)	0.013 (3)	0.002 (3)	0.004 (3)	0.000 (3)
N2	0.011 (3)	0.017 (4)	0.016 (3)	0.000 (3)	0.000 (2)	−0.002 (3)
C1	0.023 (4)	0.017 (4)	0.012 (4)	0.001 (3)	−0.002 (3)	0.005 (3)
C2	0.024 (5)	0.028 (5)	0.020 (5)	−0.002 (4)	0.003 (4)	0.001 (4)
C3	0.033 (5)	0.026 (5)	0.019 (4)	0.001 (4)	0.001 (4)	−0.003 (4)
C4	0.030 (5)	0.018 (4)	0.020 (5)	0.001 (4)	−0.001 (4)	−0.005 (3)
C5	0.025 (4)	0.015 (4)	0.018 (4)	−0.005 (4)	−0.001 (3)	−0.001 (3)
C6	0.017 (4)	0.012 (4)	0.009 (4)	0.005 (3)	0.000 (3)	0.001 (3)
C7	0.016 (4)	0.019 (4)	0.016 (4)	0.005 (3)	0.002 (3)	0.002 (3)
C8	0.020 (4)	0.014 (4)	0.019 (4)	0.000 (3)	0.003 (3)	0.004 (3)
C9	0.026 (5)	0.021 (4)	0.016 (4)	−0.004 (4)	0.008 (3)	0.000 (4)
C10	0.032 (5)	0.034 (5)	0.011 (4)	−0.002 (5)	−0.002 (4)	−0.003 (4)
C11	0.030 (5)	0.031 (5)	0.018 (5)	0.011 (4)	0.004 (4)	0.008 (4)
C12	0.027 (5)	0.015 (4)	0.022 (5)	−0.001 (3)	0.005 (4)	0.002 (3)
C13	0.015 (4)	0.014 (4)	0.023 (5)	−0.001 (3)	0.006 (3)	0.002 (3)
C14	0.028 (5)	0.016 (4)	0.017 (4)	0.001 (3)	0.001 (4)	−0.002 (3)
C15	0.030 (5)	0.025 (4)	0.017 (5)	0.004 (4)	−0.006 (4)	0.003 (4)
C16	0.030 (5)	0.025 (4)	0.020 (5)	−0.009 (4)	0.001 (4)	−0.008 (4)
C17	0.030 (5)	0.024 (4)	0.016 (4)	0.000 (4)	−0.001 (4)	−0.002 (3)
C18	0.036 (6)	0.017 (5)	0.013 (4)	0.000 (3)	−0.006 (4)	0.002 (3)
C19	0.015 (4)	0.021 (4)	0.017 (4)	−0.004 (3)	0.005 (3)	−0.002 (3)
C20	0.018 (4)	0.018 (4)	0.019 (4)	−0.001 (3)	0.001 (3)	0.006 (3)
C21	0.015 (4)	0.016 (4)	0.013 (4)	0.000 (3)	−0.002 (3)	0.004 (3)
C22	0.019 (4)	0.021 (4)	0.022 (5)	−0.002 (4)	0.001 (4)	−0.006 (4)
C23	0.022 (4)	0.034 (5)	0.019 (4)	0.004 (4)	0.006 (3)	−0.004 (4)
C24	0.027 (5)	0.030 (5)	0.015 (4)	−0.010 (4)	0.003 (4)	0.005 (4)
C25	0.018 (4)	0.020 (4)	0.022 (5)	0.000 (3)	−0.001 (4)	0.006 (4)
C26	0.009 (4)	0.020 (4)	0.019 (4)	0.001 (3)	0.004 (3)	0.000 (3)

### Geometric parameters (Å, º)

**Table:**

Cu1—S1	2.314 (2)	C5—C6	1.519 (11)
Cu1—I1	2.6467 (12)	C5—H5A	0.9900
Cu1—I2	2.6669 (12)	C5—H5B	0.9900
Cu1—I3	2.6939 (12)	C6—H6	1.0000
Cu1—Cu3	2.7444 (14)	C7—C8	1.520 (10)
Cu2—S2	2.297 (2)	C7—H7A	0.9900
Cu2—I4	2.6256 (12)	C7—H7B	0.9900
Cu2—I2	2.6544 (12)	C8—H8A	0.9900
Cu2—Cu4	2.7082 (15)	C8—H8B	0.9900
Cu2—I1	2.7230 (12)	C9—C10	1.343 (12)
Cu3—O1^i^	2.087 (6)	C9—H9	0.9500
Cu3—I3	2.6210 (12)	C10—C11	1.395 (14)
Cu3—I1	2.6458 (12)	C10—H10	0.9500
Cu3—I4^ii^	2.6833 (12)	C11—C12	1.374 (13)
Cu4—O2^iii^	2.071 (6)	C11—H11	0.9500
Cu4—I4	2.6412 (13)	C12—C13	1.415 (12)
Cu4—I3^iv^	2.6800 (13)	C12—H12	0.9500
Cu4—I2	2.7084 (13)	C14—C19	1.541 (11)
I3—Cu4^ii^	2.6800 (13)	C14—C15	1.541 (12)
I4—Cu3^iv^	2.6834 (12)	C14—H14A	0.9900
S1—C7	1.814 (8)	C14—H14B	0.9900
S1—C6	1.816 (8)	C15—C16	1.528 (13)
S2—C20	1.808 (8)	C15—H15A	0.9900
S2—C19	1.826 (9)	C15—H15B	0.9900
O1—C13	1.256 (10)	C16—C17	1.500 (12)
O1—Cu3^iii^	2.087 (6)	C16—H16A	0.9900
O2—C26	1.259 (10)	C16—H16B	0.9900
O2—Cu4^i^	2.071 (6)	C17—C18	1.535 (12)
N1—C9	1.367 (11)	C17—H17A	0.9900
N1—C13	1.395 (11)	C17—H17B	0.9900
N1—C8	1.467 (10)	C18—C19	1.525 (11)
N2—C22	1.374 (11)	C18—H18A	0.9900
N2—C26	1.391 (11)	C18—H18B	0.9900
N2—C21	1.481 (10)	C19—H19	1.0000
C1—C2	1.509 (12)	C20—C21	1.525 (11)
C1—C6	1.520 (10)	C20—H20A	0.9900
C1—H1A	0.9900	C20—H20B	0.9900
C1—H1B	0.9900	C21—H21A	0.9900
C2—C3	1.522 (13)	C21—H21B	0.9900
C2—H2A	0.9900	C22—C23	1.341 (12)
C2—H2B	0.9900	C22—H22	0.9500
C3—C4	1.535 (12)	C23—C24	1.417 (15)
C3—H3A	0.9900	C23—H23	0.9500
C3—H3B	0.9900	C24—C25	1.362 (13)
C4—C5	1.524 (11)	C24—H24	0.9500
C4—H4A	0.9900	C25—C26	1.433 (12)
C4—H4B	0.9900	C25—H25	0.9500
			
S1—Cu1—I1	108.85 (6)	C5—C6—C1	110.5 (7)
S1—Cu1—I2	118.63 (7)	C5—C6—S1	112.0 (6)
I1—Cu1—I2	106.91 (4)	C1—C6—S1	107.7 (5)
S1—Cu1—I3	97.29 (7)	C5—C6—H6	108.8
I1—Cu1—I3	116.26 (4)	C1—C6—H6	108.8
I2—Cu1—I3	109.18 (4)	S1—C6—H6	108.8
S1—Cu1—Cu3	112.03 (7)	C8—C7—S1	108.7 (5)
I1—Cu1—Cu3	58.75 (3)	C8—C7—H7A	110.0
I2—Cu1—Cu3	129.07 (5)	S1—C7—H7A	110.0
I3—Cu1—Cu3	57.62 (3)	C8—C7—H7B	110.0
S2—Cu2—I4	115.97 (7)	S1—C7—H7B	110.0
S2—Cu2—I2	108.17 (7)	H7A—C7—H7B	108.3
I4—Cu2—I2	119.84 (4)	N1—C8—C7	111.4 (6)
S2—Cu2—Cu4	134.49 (7)	N1—C8—H8A	109.4
I4—Cu2—Cu4	59.34 (4)	C7—C8—H8A	109.4
I2—Cu2—Cu4	60.66 (4)	N1—C8—H8B	109.4
S2—Cu2—I1	98.23 (6)	C7—C8—H8B	109.4
I4—Cu2—I1	106.68 (4)	H8A—C8—H8B	108.0
I2—Cu2—I1	105.09 (4)	C10—C9—N1	120.1 (9)
Cu4—Cu2—I1	127.12 (5)	C10—C9—H9	119.9
O1^i^—Cu3—I3	106.53 (17)	N1—C9—H9	119.9
O1^i^—Cu3—I1	110.92 (18)	C9—C10—C11	121.0 (9)
I3—Cu3—I1	118.90 (4)	C9—C10—H10	119.5
O1^i^—Cu3—I4^ii^	106.23 (18)	C11—C10—H10	119.5
I3—Cu3—I4^ii^	105.84 (4)	C12—C11—C10	118.7 (9)
I1—Cu3—I4^ii^	107.65 (4)	C12—C11—H11	120.7
O1^i^—Cu3—Cu1	132.47 (18)	C10—C11—H11	120.7
I3—Cu3—Cu1	60.23 (3)	C11—C12—C13	122.0 (8)
I1—Cu3—Cu1	58.78 (3)	C11—C12—H12	119.0
I4^ii^—Cu3—Cu1	121.23 (5)	C13—C12—H12	119.0
O2^iii^—Cu4—I4	106.57 (17)	O1—C13—N1	117.8 (8)
O2^iii^—Cu4—I3^iv^	120.84 (17)	O1—C13—C12	126.5 (7)
I4—Cu4—I3^iv^	105.37 (4)	N1—C13—C12	115.6 (8)
O2^iii^—Cu4—Cu2	121.72 (17)	C19—C14—C15	110.6 (7)
I4—Cu4—Cu2	58.77 (3)	C19—C14—H14A	109.5
I3^iv^—Cu4—Cu2	117.37 (5)	C15—C14—H14A	109.5
O2^iii^—Cu4—I2	101.65 (17)	C19—C14—H14B	109.5
I4—Cu4—I2	117.31 (4)	C15—C14—H14B	109.5
I3^iv^—Cu4—I2	105.88 (4)	H14A—C14—H14B	108.1
Cu2—Cu4—I2	58.69 (4)	C16—C15—C14	110.0 (8)
Cu3—I1—Cu1	62.47 (3)	C16—C15—H15A	109.7
Cu3—I1—Cu2	107.56 (4)	C14—C15—H15A	109.7
Cu1—I1—Cu2	73.38 (4)	C16—C15—H15B	109.7
Cu2—I2—Cu1	74.17 (4)	C14—C15—H15B	109.7
Cu2—I2—Cu4	60.65 (3)	H15A—C15—H15B	108.2
Cu1—I2—Cu4	113.57 (4)	C17—C16—C15	112.1 (8)
Cu3—I3—Cu4^ii^	74.12 (4)	C17—C16—H16A	109.2
Cu3—I3—Cu1	62.16 (4)	C15—C16—H16A	109.2
Cu4^ii^—I3—Cu1	99.33 (4)	C17—C16—H16B	109.2
Cu2—I4—Cu4	61.89 (4)	C15—C16—H16B	109.2
Cu2—I4—Cu3^iv^	107.19 (4)	H16A—C16—H16B	107.9
Cu4—I4—Cu3^iv^	73.74 (4)	C16—C17—C18	112.0 (8)
C7—S1—C6	103.4 (4)	C16—C17—H17A	109.2
C7—S1—Cu1	106.5 (3)	C18—C17—H17A	109.2
C6—S1—Cu1	110.8 (3)	C16—C17—H17B	109.2
C20—S2—C19	104.0 (4)	C18—C17—H17B	109.2
C20—S2—Cu2	109.5 (3)	H17A—C17—H17B	107.9
C19—S2—Cu2	111.5 (3)	C19—C18—C17	110.5 (8)
C13—O1—Cu3^iii^	127.4 (5)	C19—C18—H18A	109.5
C26—O2—Cu4^i^	126.2 (5)	C17—C18—H18A	109.5
C9—N1—C13	122.6 (8)	C19—C18—H18B	109.5
C9—N1—C8	119.6 (8)	C17—C18—H18B	109.5
C13—N1—C8	117.8 (7)	H18A—C18—H18B	108.1
C22—N2—C26	122.1 (7)	C18—C19—C14	110.9 (7)
C22—N2—C21	119.3 (7)	C18—C19—S2	111.6 (7)
C26—N2—C21	118.6 (7)	C14—C19—S2	105.6 (6)
C2—C1—C6	111.1 (7)	C18—C19—H19	109.6
C2—C1—H1A	109.4	C14—C19—H19	109.6
C6—C1—H1A	109.4	S2—C19—H19	109.6
C2—C1—H1B	109.4	C21—C20—S2	114.5 (6)
C6—C1—H1B	109.4	C21—C20—H20A	108.6
H1A—C1—H1B	108.0	S2—C20—H20A	108.6
C1—C2—C3	111.3 (7)	C21—C20—H20B	108.6
C1—C2—H2A	109.4	S2—C20—H20B	108.6
C3—C2—H2A	109.4	H20A—C20—H20B	107.6
C1—C2—H2B	109.4	N2—C21—C20	108.9 (6)
C3—C2—H2B	109.4	N2—C21—H21A	109.9
H2A—C2—H2B	108.0	C20—C21—H21A	109.9
C2—C3—C4	110.3 (7)	N2—C21—H21B	109.9
C2—C3—H3A	109.6	C20—C21—H21B	109.9
C4—C3—H3A	109.6	H21A—C21—H21B	108.3
C2—C3—H3B	109.6	C23—C22—N2	121.5 (9)
C4—C3—H3B	109.6	C23—C22—H22	119.2
H3A—C3—H3B	108.1	N2—C22—H22	119.2
C5—C4—C3	111.3 (7)	C22—C23—C24	119.1 (9)
C5—C4—H4A	109.4	C22—C23—H23	120.4
C3—C4—H4A	109.4	C24—C23—H23	120.4
C5—C4—H4B	109.4	C25—C24—C23	120.1 (8)
C3—C4—H4B	109.4	C25—C24—H24	120.0
H4A—C4—H4B	108.0	C23—C24—H24	120.0
C6—C5—C4	111.1 (7)	C24—C25—C26	121.2 (8)
C6—C5—H5A	109.4	C24—C25—H25	119.4
C4—C5—H5A	109.4	C26—C25—H25	119.4
C6—C5—H5B	109.4	O2—C26—N2	119.0 (7)
C4—C5—H5B	109.4	O2—C26—C25	125.0 (8)
H5A—C5—H5B	108.0	N2—C26—C25	116.0 (7)
			
C6—C1—C2—C3	57.6 (10)	C19—C14—C15—C16	56.2 (10)
C1—C2—C3—C4	−56.0 (10)	C14—C15—C16—C17	−55.8 (11)
C2—C3—C4—C5	54.9 (10)	C15—C16—C17—C18	55.6 (11)
C3—C4—C5—C6	−55.4 (10)	C16—C17—C18—C19	−55.2 (11)
C4—C5—C6—C1	56.1 (9)	C17—C18—C19—C14	55.8 (10)
C4—C5—C6—S1	176.2 (6)	C17—C18—C19—S2	173.2 (6)
C2—C1—C6—C5	−57.2 (9)	C15—C14—C19—C18	−57.1 (10)
C2—C1—C6—S1	−179.8 (6)	C15—C14—C19—S2	−178.1 (6)
C7—S1—C6—C5	63.5 (6)	C20—S2—C19—C18	58.8 (7)
Cu1—S1—C6—C5	177.3 (5)	Cu2—S2—C19—C18	176.7 (6)
C7—S1—C6—C1	−174.7 (6)	C20—S2—C19—C14	179.3 (5)
Cu1—S1—C6—C1	−60.9 (6)	Cu2—S2—C19—C14	−62.8 (6)
C6—S1—C7—C8	−154.7 (6)	C19—S2—C20—C21	62.3 (7)
Cu1—S1—C7—C8	88.4 (6)	Cu2—S2—C20—C21	−57.0 (6)
C9—N1—C8—C7	104.3 (8)	C22—N2—C21—C20	−101.2 (8)
C13—N1—C8—C7	−77.0 (9)	C26—N2—C21—C20	76.9 (8)
S1—C7—C8—N1	−179.3 (6)	S2—C20—C21—N2	174.1 (5)
C13—N1—C9—C10	−1.3 (13)	C26—N2—C22—C23	1.4 (12)
C8—N1—C9—C10	177.3 (8)	C21—N2—C22—C23	179.4 (8)
N1—C9—C10—C11	0.4 (14)	N2—C22—C23—C24	−0.1 (13)
C9—C10—C11—C12	0.2 (15)	C22—C23—C24—C25	0.0 (13)
C10—C11—C12—C13	0.1 (14)	C23—C24—C25—C26	−1.2 (13)
Cu3^iii^—O1—C13—N1	165.9 (5)	Cu4^i^—O2—C26—N2	−179.4 (5)
Cu3^iii^—O1—C13—C12	−15.3 (13)	Cu4^i^—O2—C26—C25	1.9 (12)
C9—N1—C13—O1	−179.5 (7)	C22—N2—C26—O2	178.8 (7)
C8—N1—C13—O1	1.9 (11)	C21—N2—C26—O2	0.8 (10)
C9—N1—C13—C12	1.6 (12)	C22—N2—C26—C25	−2.4 (11)
C8—N1—C13—C12	−177.1 (7)	C21—N2—C26—C25	179.6 (7)
C11—C12—C13—O1	−179.8 (9)	C24—C25—C26—O2	−179.0 (8)
C11—C12—C13—N1	−0.9 (13)	C24—C25—C26—N2	2.3 (12)

Symmetry codes: (i) *x*, *y*+1, *z*; (ii) *x*−1, *y*, *z*; (iii) *x*, *y*−1, *z*; (iv) *x*+1, *y*, *z*.

### Hydrogen-bond geometry (Å, º)

Cg1 is the centroid of the N2/C22–C26 ring

**Table:**

*D*—H···*A*	*D*—H	H···*A*	*D*···*A*	*D*—H···*A*
C8—H8*A*···I4^ii^	0.99	3.26	4.107 (8)	145
C12—H12···I1^iii^	0.95	3.30	3.899 (8)	123
C21—H21*B*···I3^iv^	0.99	3.08	3.923 (8)	144
C5—H5*A*···*Cg*1^v^	0.99	3.00	3.948 (9)	162

Symmetry codes: (ii) *x*−1, *y*, *z*; (iii) *x*, *y*−1, *z*; (iv) *x*+1, *y*, *z*; (v) *x*−1, *y*−1, *z*.
